# Audit of Rising Rates of Methamphetamine-Induced Psychosis (MIP): Assessment and Management in an Inpatient Addiction Ward, Allied Hospital II, Faisalabad.

**DOI:** 10.1192/bjo.2026.11570

**Published:** 2026-06-30

**Authors:** Sammar Fatima, Imtiaz Ahmad Dogar, Duaa Fatima, Asadullah Raoufy, Sinha Tahir

**Affiliations:** Faisalabad Medical University, Faisalabad, Pakistan

## Abstract

**Aims:**

Methamphetamine-induced psychosis (MIP) is recognised in both ICD-11 and DSM-5-TR as a substance-induced psychotic disorder which is associated with severe agitation, violence risk, and medical complications. In Pakistan, MIP presentations to inpatient addiction services have increased recently, however, data on service impact and adherence to best-practice standards remain limited. This audit aims to describe monthly trends and proportions of MIP admissions among all inpatient addiction ward admissions, and to evaluate compliance with international best-practice standards for assessment and management of MIP, in order to identify areas for quality improvement.

**Methods:**

A mixed-method clinical audit was conducted in the inpatient addiction ward at Allied Hospital II, Faisalabad. A descriptive service-activity review examined all inpatient addiction admissions over a six-month period (01 July–31 December 2025) to quantify monthly MIP admissions. Subsequently, an admission-based case-note audit assessed consecutive MIP admissions against predefined standards derived from NICE guidance, the Maudsley Prescribing Guidelines, and international recommendations. Adults (≥18 years) admitted with a clinical diagnosis of MIP were included. Admissions with primary psychotic disorders, delirium, psychosis attributable to another substance, or incomplete documentation were excluded. Data were extracted from clinical notes using a structured audit tool.

**Results:**

During the audit period, 82 inpatient addiction admissions were recorded, of which 19 (23.2%) were for MIP. Monthly MIP admissions ranged from 2 to 4 cases, representing 18.2% to 33.3% of total admissions, indicating a sustained and clinically significant service burden. In the standards audit (n=19), documentation of stimulant use history (94.7%), temporal relationship between methamphetamine use and psychotic symptoms (89.5%), exclusion of delirium or medical causes (100%), baseline physical observations (94.7%), and mental state examination (94.7%) met audit targets. Prescribing details and adverse-effect documentation were complete in all cases (100%). Areas of lower compliance included objective toxicology confirmation (42.1%), baseline blood glucose measurement (52.6%), structured risk assessment (78.9%), documentation of de-escalation prior to medication (73.7%), medication safety checks for contraindications or interactions (78.9%), post-medication monitoring (84.2%), and timely reassessment following intervention (73.7%).

**Conclusion:**

Methamphetamine-induced psychosis accounted for nearly one-quarter of inpatient addiction admissions over six months, highlighting a substantial and ongoing clinical burden. While core diagnostic assessment and prescribing practices were generally well documented, important gaps were identified in toxicology confirmation, metabolic screening, structured risk assessment, and post-intervention monitoring. These findings informed the development of a standardized MIP admission checklist and targeted staff education, with re-audit planned to assess the impact of these quality improvement interventions.

